# Efficacy and safety of Lianhua Qingwen combined with conventional antiviral Western Medicine in the treatment of coronavirus disease (covid-19) in 2019: Protocol for a systematic review and meta-analysis

**DOI:** 10.1097/MD.0000000000021404

**Published:** 2020-07-24

**Authors:** Xiaolin Zhang, Di Cao, Junnan Liu, Qi Zhang, Mingjun Liu

**Affiliations:** a Changchun University of Chinese Medicine; b Changchun Hospital of Chinese Medicine; c Department of lung diseases, the Third Clinical Hospital of Changchun University of Chinese Medicine, Changchun, Jilin, China.

**Keywords:** COVID-19, Lianhua Qingwen, meta-analysis, protocol, systematic review

## Abstract

**Background:**

The novel coronavirus pneumonia (COVID-19) has spread to >200 countries and regions. There is no effective antiviral drug for COVID-19. Traditional Chinese medicine, such as Lianhua Qingwen, has achieved some curative effect in many countries, but its effect is not clear. We aim to assess the efficacy and safety of Lianhua Qingwen combined with Conventional antiviral Western Medicine in Clinical treatment of COVID-19 or asymptomatic infection.

**Methods:**

The following electronic bibliographic databases will be searched to identify relevant studies: CNKI, CBM, VIP and Wanfang databases, PubMed, EMBASE, MEDLINE, Cochrane central, and clinical trial registration centers, such as China Clinical Trial Registration Center (ChiCTR), Netherlands National Trial Registration Center (NTR) and clinical trials.gov. In addition, Manual retrieval of articles, conference papers, ongoing experiments, internal reports, among others, to supplement electronic retrieval. Select all eligible studies published before May 8, 2020.

According to the Cochrane Handbook “bias risk” assessment tool, bias risk is independently assessed. The independent Newcastle Ottawa scale was used to conduct methodological quality assessment of nonrandomized trials. STATA15.1 and RevMan5.3 software were used to analyze meta outcomes of different intervention measures for the treatment of new crown pneumonia and the control group (conventional antiviral western medicine treatment) clinical efficacy.

**Results:**

This study will provide a relatively high-quality synthesis of current evidence of Lianhua Qingwen combined with Conventional antiviral Western Medicine in the treatment of COVID-19 from several aspects including the Clinical effective rate, CT improvement rate, severe conversion rate, antipyretic time, disappearance rate of fever symptoms, disappearance rate of cough symptoms, disappearance rate of asthenia symptoms, and adverse drug events.

**Conclusion:**

The conclusion of this review will provide evidence to judge whether Lianhua Qingwen combined with Conventional antiviral Western Medicine is an effective and safe intervention for COVID-19.

**Ethics and dissemination:**

This systemic review will evaluate the efficacy and safety of Lianhua Qingwen combined with Conventional antiviral Western Medicine in the treatment of COVID-19. Since all the data included are published, the systematic review does not need ethical approval.

**INPLASY registration number:**

INPLASY202060067

## 1 Introduction

Recently, the novel coronavirus pneumonia (COVID-19) epidemic has spread continuously and has been classified as class B infectious diseases (class a management).^[1]^ Over 200 countries and regions in the world have reported the confirmed cases successively, and become the global fast spread pandemic.^[2]^ The main manifestations of the disease are fever, fatigue, and dry cough. A few patients have cough, expectoration, chest distress, dyspnea, muscle ache, diarrhea, and other symptoms.^[3]^ It is reported that 4,430,100 cases have been confirmed in the world, and 299,451 cases have died. The proportion of severe patients admitted to covid-19 hospital is 15.7%, and the death rate of confirmed patients is 6.7%.^[4]^ However, at present, there is no effective antiviral treatment drug confirmed for covid-19.^[5–9]^ In the early stage, there is no specific medicine or vaccine; the intervention of Chinese traditional medicine compound Lianhua Qingwen and other traditional Chinese medicine have played an important role.^[10–14]^ Data showed that novel coronavirus pneumonia was confirmed in China, and 74,187 people used Chinese medicine, accounting for 91.5%, of which 61,449 in Hubei Province used Chinese medicine, accounting for 90.6%. Clinical observation shows that the total effective rate of traditional Chinese medicine is >90%.^[15]^

Meanwhile, China also provides technical solutions through long-distance video exchanges to share treatment experience with Japan, South Korea, Italy, the United States, Iran, Singapore, and so on. The 3 drugs and 3 parties in China have been effective in many countries around the world, and once again reveal the special advantages of the Chinese herbal medicine “overall adjustment and multi target therapy,” which suggests the application of Lianhua Qingwen granule. It has important clinical application value to treat patients with covid-19.

Lianhua Qingwen capsule (granule) is an Innovative Patent Chinese medicine for the treatment of influenza, which was developed by the green channel of the new drug approval during the SARS period in 2003 based on the pathology of collateral of traditional Chinese medicine. It has a wide-spectrum antiviral, effective antibacterial, antipyretic and anti-inflammatory, antitussive and expectorant, immune-regulating and other systemic intervention functions,^[16–25]^ especially for the coronavirus SARS^[26]^ And Middle East respiratory syndrome (MERS)^[27]^ have significant inhibitory and killing effects.

The Chinese medicine compound Lianhua Qingwen has been listed as the recommended drug for the diagnosis and treatment of respiratory infectious diseases by the national Wei Jian administration and the State Administration of traditional Chinese medicine for 20 times. It has become a representative Chinese patent medicine for public health emergencies of the respiratory system. It has been recommended as a recommended drug for the diagnosis and treatment of pneumonia caused by a new coronavirus (which has been included in the fifth, sixth, and seventh edition of the diagnostic and therapeutic plan version).

However, there are “individual differences” in the efficacy of traditional Chinese medicine compound Lianhua Qingwen, which makes the quality of evidence of clinical efficacy of traditional Chinese medicine low. Therefore, it is necessary to carry out rigorous and objective quality evaluation for different types of clinical research, and the effectiveness analysis results obtained on this basis are more convincing. Novel coronavirus pneumonia is a new method for the treatment of new crown pneumonia. It is based on the published clinical research literature at present stage. It is to systematically understand the curative effect of Lianhua Qingwen on the treatment of new crown pneumonia, and provide a reference for the design and follow-up work of the Chinese medicine compound Lianhua Qingwen in the global epidemic prevention and clinical trial design and follow-up work.

## 2 Methods

### 2.1 Inclusion criteria for study selection

#### 2.1.1 Types of studies

All randomized controlled trials (RCTs), Retrospective cohort study (RCSs), Comparison before and after the study (BAs) of Lianhua Qingwen combined with Conventional antiviral Western Medicine for COVID-19 will be included. Excluded from the meta-analysis are duplicated publications; the control group also used traditional Chinese medicine intervention, studies with unavailable or incorrect data, articles not reporting outcomes of interest. Also excluded are studies enrolling <30 participants.

#### 2.1.2 Types of patients

Patients diagnosed with COVID-19 of all ages and racial groups will be included. All patients must be diagnosed with prediabetes by clearly defined or internationally recognized criteria.

#### 2.1.3 Types of interventions

Exclusion: studies using two or more Chinese herbal compound as intervention measures will be excluded.

#### 2.1.4 Types of outcome measures

##### 2.1.4.1 The primary out-comes

The clinical effective rate, CT improvement rate, and severe conversion rate will be assessed as the primary outcomes.

##### 2.1.4.2 The secondary outcomes

The secondary outcomes of this review will include antipyretic time, disappearance rate of fever symptoms, disappearance rate of cough symptoms, disappearance rate of asthenia symptoms, and adverse drug events.

### 2.2 Search methods for the identification of studies

System searches CNKI, CBM, VIP and Wanfang databases, PubMed, EMBASE, MEDLINE, Cochrane central, and clinical trial registration centers, such as ChiCTR, NTR and clinical Trials.gov.

In addition, Manual retrieval of articles, conference papers, ongoing experiments, internal reports, among others, to supplement electronic retrieval. Select all eligible studies published before May 8, 2020.

In PubMed, MEDLINE, and EMBASE, search with “Lianhua Qingwen” and “COVID-19” as subject words. In WANFANG, novel coronavirus pneumonia, 2019-nCOV, COVID-19, and SARS-COV-2 were first searched for the keywords, then searched for the theme word “Lianhua Qingwen” and “Lianhua Qingke.” The 2 search were merged with the “relationship.” Novel coronavirus pneumonia is being set up by CNKI for the new research project. Because the epidemic situation is still ongoing, there are still clinical studies in progress. Before the evaluation results of this system are published, all databases will be retrieved again. If there are latest published studies, they will be directly included in the comprehensive treatment together with previous studies.

### 2.3 Data collection and analysis

#### 2.3.1 Selection of studies

Records from databases and other resources will be uploaded to a database created by EndNote9.7 software. The abstracts of all studies will be independently screened by the review authors (XLZand QL). The full text of articles potentially suitable for the review will be obtained for further assessing eligibility based on the inclusion criteria or/and exclusion criteria. The studies that do not fulfill the inclusion criteria will be excluded and listed with reasons for their exclusion. Any disagreement will be resolved by consensus or discussion with a 3rd party (MJL).

#### 2.3.2 Data extraction and management

Two reviewers (XLZ and JNL) will assess the eligibility of the studies retrieved during the searches independently using the inclusion and exclusion criteria. The following data will then be extracted from the studies selected for inclusion using a data collection form, and recorded onto an Excel file: first author and year, study design, sample, intervention, type of measures, risk of bias assessment, and findings. The results will be cross-checked by the 2 reviewers, and any disagreements will be resolved by consensus, with any ongoing differences in opinion being arbitrated by a third reviewer (LMJ).

We may also contact the original authors to provide additional relevant information, if necessary.

The data extraction form will include the following items:

1. general information: title, authors, year of publication, and Study area, average age, average course of disease and treatment time;
2. trial characteristics: design, duration of follow-up, method of randomization, allocation concealment, incomplete outcome data, blinding (patients, people administering treatment, outcome assessors);
3. intervention(s): intervention(s) (The dosage form of Lianhua Qingwen, Antiviral Western Medicine, Number and dose of oral administration, duration of session), comparison intervention(s) (Antiviral Western Medicine, Number and dose of oral administration, duration of session);
4. patients: total number and number in both groups, baseline characteristics, diagnostic criteria, withdrawals, and losses to follow-up (reasons, description);
5. outcomes: outcomes specified above, adverse drug reactions, and adverse time, length of follow-up, quality of reporting of outcomes.

#### 2.3.3 Assessment of risk of bias in included studies

Two reviewers/authors (ZXL and ZQ) will independently evaluate the quality of the included trials through assessing the risk of bias using the following tools, when appropriate:

1. For RCTs, the Cochrane Collaboration's tool for assessing risk of bias will be used;^[28]^
2. For NRIs, evaluate its methodological quality with the Newcastle Ottawa scale (NOS).^[29]^

The following aspects of included trials will be assessed: sequence generation, allocation concealment, blinding of participants, personnel and outcome assessors, incomplete outcome data, and selective outcome reporting.

We will judge the each domain as “low risk of bias,” “high risk of bias," or “uncertain risk of bias" according to Higgins (2011), and pay particular attention to the risk of bias of cluster-randomised trials. Also illustrates the potential biases within each of the included studies by presenting a “risk of bias," table, graph, and summary.

#### 2.3.4 Measures of treatment effect

Dichotomous outcomes will be presented as risk ratio and 95% confidence intervals (CIs), and continuous outcomes will be presented as mean difference/standard mean difference and 95% CIs.

**S**tatistical analysis was conducted by Revman 5.3 software. Risk ratio (RR) was used to express the severe conversion rate and CT improvement rate absorption rate; mean difference (MD) was used to express the time of fever reduction in secondary indexes; Odds Ratio (OR) was used to express clinical efficiency and symptom disappearance rate.

Data synthesis reports the intervention effect of the original study by integrating the cohort study. At the same time, the single arm study was included in the analysis, not involved in data synthesis, only used to assist in displaying the clinical characteristics of the subjects, not for efficacy evaluation. After determining how to combine different types of studies, NRIs use the method of inverse variance of random-effect model to evaluate and calculate the 95% CI of the effect value.

#### 2.3.5 Assessment of heterogeneity

Statistical heterogeneity will be assessed with the *I*^2^ statistic.^[30]^ The *I*^2^ statistic of <50% indicates a low level of statistical heterogeneity, and that of ≥50% will be considered substantial statistical heterogeneity. If substantial heterogeneity is identified, we will report it and explore possible causes using sensitivity analysis and subgroup analysis.

#### 2.3.6 Assessment of reporting biases

A funnel plot will be constructed and examined to assess publication bias and possible small study biases if the group include more than 10 trials.^[31]^ The results will be interpreted carefully based on several explanations for funnel plot asymmetry.

#### 2.3.7 Data synthesis

If ≥2 eligible RCTs are identified, meta-analysis will be performed with Review Manager 5.3. All tests are 2-tailed, and *P* < .05 is considered statistically significant. Whether a fixed-effects or a random-effects model will be used depends on the results of *I*^2^ test for heterogeneity. If *I*^2^ test <50%, a fixed-effects model will be used to pool the data. If *I*^2^ test ≥50%, a random-effects model will be used for data analysis instead. Subgroup analysis and sensitivity analysis will be performed to explore the causes of heterogeneity. If meta-analysis is not applicable, we will conduct a systematic narrative synthesis providing information to summarize and explain the characteristics and findings of the included studies.

#### 2.3.8 Subgroup analysis

We plan to carry out the following subgroup analyses if possible: the study area is different, the average course of disease is different, and the length of treatment is different. We will use the formal test for subgroup interactions in Review Manager 5.3.

#### 2.3.9 Sensitivity analysis

We will perform the sensitivity analysis to explore the effects of trial risk of bias on primary outcomes if possible. In the analysis, we will exclude lower-quality trials and repeat the meta-analyses to examine whether the quality of included studies influences the pooled results.

#### 2.3.10 Grading the quality of evidence

The Grading of Recommendations Assessment, Development, and Evaluation (GRADE) methodology^[32]^ will be used to assess the quality of the evidence and risk of bias with on-line GRADE (https://www.gradeworkinggroup.org/). The assessment will be adjudicated into 4 levels: high, moderate, low, or very low.

## 3 Discussion

**Figure 1:**
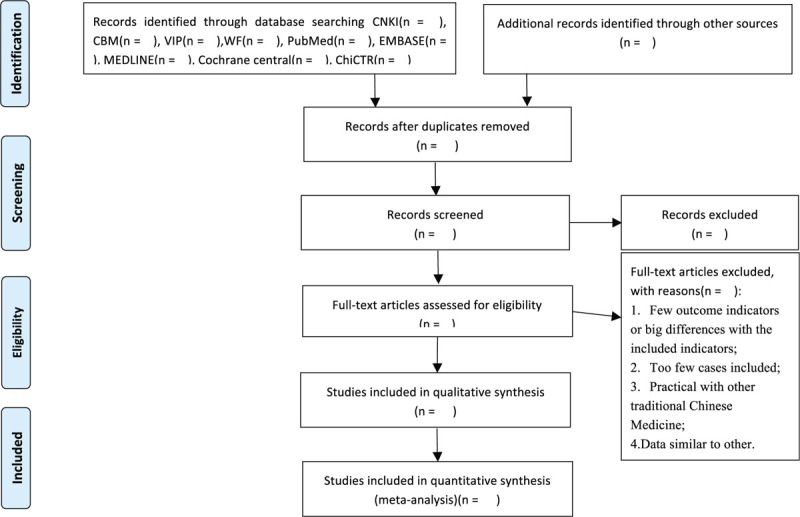
PRISMA flow diagram of study and exclusion.

Lianhua Qingwen formula is based on the theory of collateral disease, to reveal the law of transmission of respiratory infectious diseases caused by virus. The treatment strategy of “active intervention” is put forward, which includes the treatment of both exterior and interior, the treatment of syndrome first, the treatment of disease cutting off, the overall adjustment, and multitarget treatment. It was created on the basis of establishing the treatment method of “clearing away plague and detoxification, releasing lung and heat.”^[33]^ Based on treatise on Maxingshigan Decoction of “Febrile Diseases” and yinqiao powder of “Differentiation of febrile diseases,”, the prescriptions are composed to protect Qi and treat together, promote lung, and relieve heat. In addition, we learned from the experience of Rhubarb in the treatise on “Febrile Diseases” in the treatment of epidemic diseases. We first used Rhubarb to cure the disease, dredge the internal organs and relieve the heat, and then made Huoxiang fragrant and moistening. It is compatible with Rhodiola to clear lung and remove blood stasis. All the prescriptions are used to clear the toxic heat in the lung, clear the lung qi, and cut off the disease. The composition of Lianhua Qingwen granules is consistent with the pathogenesis of the disease. It embodies the active therapeutic principles of clearing away plague and detoxification, releasing lung heat, and aromatizing dampness.^[33]^ Previous pharmacodynamic studies have confirmed that Lianhua Qingwen capsule can significantly inhibit the SARS CoV virus in cultured cells in vitro, and has a significant inhibitory effect on H1N1, H3N2, h7n9, and other influenza viruses,^[34–36]^ indicating that Lianhua Qingwen capsule has played an important role in the prevention and control of public health events of respiratory system caused by virus. The COVID-19 may improve the clinical symptoms associated with fever, cough, and fatigue. It has important clinical significance for alleviating the severity of COVID-19 and shortening the course of disease. There are an increasing number of studies on for COVID-19 published with inconclusive results in recent. Lianhua Qingwen combined with conventional antiviral Western Medicine may be a useful treatment for prediabetes. Therefore, it is essential to perform a systematic review and meta-analysis to assess the effect of Lianhua Qingwen combined with conventional antiviral Western Medicine in clinical treatment of COVID-19 or asymptomatic infection. The flow diagram of this systematic review is shown in Figure 1.

This review will be helpful to clinicians treating COVID-19 and asymptomatic infection may provide evidence for researchers. Before the successful development of targeted vaccine, >4 million patients with covid-19 and asymptomatic infection around the world may also benefit from traditional Chinese medicine such as Lianhua Qingwen.

However, this systematic review will have some limitations. Because the novel coronavirus pneumonia has not yet been completely subsided, more large-sample clinical studies are still ongoing. Therefore, the completeness of the evidence is not yet available. In addition, only studies published in English and Chinese will be searched and included because of language barriers, so a language bias may exist. High heterogeneity may also arise from the various evaluations. Nevertheless, this systematic view should help further expand our understanding of Lianhua Qingwen combined with conventional antiviral Western Medicine treatments in COVID-19 and asymptomatic infection.

The PRISMA-P (Preferred Reporting Items for Systematic review and Meta-Analysis Protocols) checklist of this protocol is presented in PRISMA-P checklist.

## 4 Statement

It is not necessary for ethical approval because this article is based on previously conducted studies and does not involve any new studies of human or animal subjects performed by any of the authors. The protocol will be disseminated in a peer-reviewed journal or presented at a relevant conference.

All relevant data are within the article and its supporting information files.

## Author contributions

XLZ conceived of the study, and perform this review. The manuscript was drafted by XLZ and MJL. DC and QZ developed the search strategy. XLZ and QZ will independently screen the potential studies and extract data. XLZ and JNL will assess the risk of bias and perform data synthesis. MJL will arbitrate any disagreement and ensure that no errors occur during the review. All review authors critically reviewed, revised and approved the subsequent and final version of the protocol.

**Conceptualization:** Mingjun Liu.

**Data curation:** Xiaolin Zhang, Di Cao, Qi Zhang, Junnan Liu.

**Methodology:** Xiaolin Zhang.

**Project administration:** Xiaolin Zhang.

**Supervision:** Mingjun Liu.

**Writing – original draft:** Xiaolin Zhang, Di Cao.

**Writing – review & editing:** Xiaolin Zhang.
